# Clinical validation of the self-rated 6-item Hamilton Depression Rating Scale among inpatients

**DOI:** 10.1192/j.eurpsy.2022.690

**Published:** 2022-09-01

**Authors:** P. Kølbæk, S.D. Østergaard

**Affiliations:** 1 Aarhus University Hospital - Psychiatry, Department Of Affective Disorders, Aarhus N, Denmark; 2 Aarhus University, Department Of Clinical Medicine, Aarhus N, Denmark

**Keywords:** Psychiatric Status Rating Scales, Psychometrics, Depression, Surveys and Questionnaires

## Abstract

**Introduction:**

Measurement-based care (i.e., the systematic use of rating scales to guide clinical decision-making) has shown great promise in the treatment of major depression in clinical trials. Unfortunately, measurement-based care has not yet gained ground in clinical practice, possibly because clinician-rated scales are time-consuming and limited by the availability of trained raters. Hence, brief and valid self-rated scales (questionnaires) may serve as an alternative or supplement to clinician-rated scales. The self-rated 6-item Hamilton Depression Rating Scale (HAM-D6-SR) has shown some promise in this regard, but its validity among inpatients remains unclear.

**Objectives:**

The objective of this study is to evaluate the criterion validity and responsiveness (sensitivity to change) of the HAM-D6-SR among inpatients using the clinician-rated 17-item Hamilton Rating Scale for Depression (HAM-D17) as gold standard reference.

**Methods:**

Inpatients with depression will complete the HAM-D6-SR twice during admission (at least one week between the two self-ratings). At both occasions, the patients will subsequently be rated on the HAM-D17 by trained raters, who are blind to the HAM-D6-SR ratings. The agreement between the HAM-D6-SR and the HAM-D6 extracted from the HAM-D17 will be evaluated using intra-class correlation.

**Results:**

A total of 100 inpatients will be recruited for the study. Data collection is ongoing, and the results of the study will be presented at the 2022 EPA meeting.

**Conclusions:**

If the agreement between the HAM-D6-SR and the HAM-D6 extracted from the HAM-D17 is satisfactory, the HAM-D6-SR could inform decision-making in the treatment of depression.

**Disclosure:**

The presenting author, PK, declares no conflict of interests. Co-author, SDØ, has received the 2020 Lundbeck Foundation Young Investigator Prize. Furthermore, SDØ owns units of mutual funds with stock tickers DKIGI, DKIDKIX, MAJGRO, NBIDE, SPVILRKL and WE

